# Three-View Relative Pose Estimation Under Planar Motion Constraints

**DOI:** 10.3390/vision9030072

**Published:** 2025-08-25

**Authors:** Ziqin Dai, Weimin Lv, Liang Liu

**Affiliations:** Naval Aviation University, Yantai 264001, China; daiziqin_001@163.com (Z.D.); liul513@126.com (L.L.)

**Keywords:** visual localization, relative pose estimation, three-view geometry

## Abstract

Vision-based relative pose estimation serves as a core technology for high-precision localization in autonomous vehicles and mobile platforms. To overcome the limitations of conventional three-view pose estimation methods that rely heavily on dense feature matching and incur high computational costs, this paper proposes an efficient three-point correspondence algorithm based on planar motion constraints. The method constructs trifocal tensor constraint equations and develops a linearized three-point solution framework, enabling rapid relative pose estimation using merely three corresponding points in three views. In simulation experiments, we systematically analyzed the robustness of the algorithm under complex conditions that included image noise, angular deviation, and vibration. The method was further validated in real-world scenarios using the KITTI public dataset. Experimental results demonstrate that under the condition of satisfying the planar motion assumption, the proposed method achieves significantly improved computational efficiency compared with traditional methods (including general three-view methods, two-view planar motion estimation methods, and classical two-view methods), with the single-solution time reduced by more than 80% compared to general three-view methods. In the public dataset, our algorithm achieves a median rotation estimation error of less than 0.0545 degrees and maintains a translation estimation error of less than 2.1319 degrees. The proposed method exhibits higher computational efficiency and better numerical stability compared to conventional algorithms. This research provides an effective pose estimation solution with real-time performance and high accuracy for planar motion platforms such as autonomous vehicles and indoor mobile robots, demonstrating substantial engineering application value.

## 1. Introduction

For moving platforms, such as drones and unmanned vehicles, using the sensors installed on them to estimate their position and attitude is a prerequisite to meet the application needs [1]. Traditional pose estimation methods primarily rely on Global Navigation Satellite Systems (GNSS) and Inertial Measurement Units (IMUs). However, in indoor environments, urban canyons, or complex electromagnetic conditions, GNSS signals are susceptible to multipath effects, non-line-of-sight reception, and even spoofing interference [2,3]. Vision-based pose estimation methods have attracted significant research attention in recent years as an effective complementary solution to improve localization reliability, due to their non-contact nature, high accuracy, and low cost [4]. However, visual pose estimation still faces many challenges. Especially in complex, dynamic scenarios, efficiently and robustly solving the relative pose of the camera has become a research hotspot.

**Figure 1:**
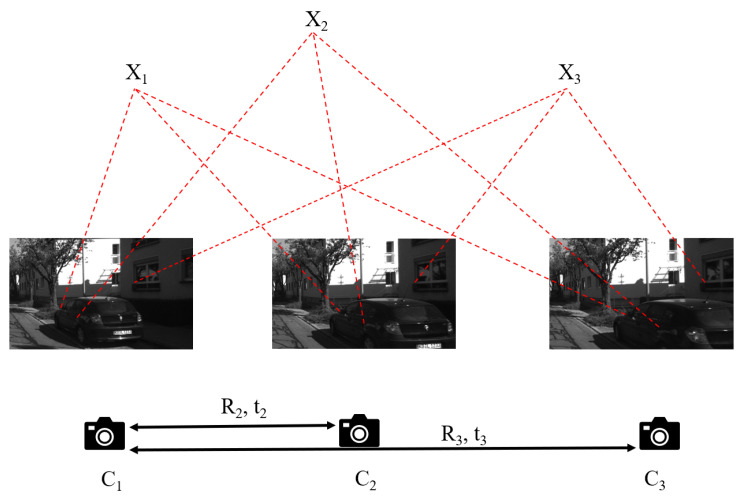
Estimation of the three-view pose in planar motion.

In the vision-based relative pose estimation method, the technology for estimating relative pose between two views is already very mature. However, the presence of mismatches frequently leads to erroneous pose solutions, making the estimation process prone to degenerate cases. In contrast, the three-view method introduces additional geometric constraints that effectively resolve correspondence ambiguity and improve the robustness of pose estimation [5]. Notably, in many practical applications (e.g., urban road navigation, indoor mobile robots), the platform motion often satisfies the planar motion assumption. This assumption reduces the 6-DoF pose problem to a 3-DoF problem, significantly simplifying the solution. Therefore, this paper proposes a three-view pose estimation method based on three-point correspondences by exploiting planar motion constraints. The method establishes linear equations through the trifocal tensor and efficiently solves for rotation and translation parameters using SVD decomposition, as illustrated in Figure 1. The main contributions of this paper are as follows:

- **Theoretical innovation:** By integrating planar motion constraints with trifocal tensor theory, we derive a linear solver requiring only three point correspondences, eliminating the dependency on dense feature points in conventional methods. This theoretical framework reduces the 6-DoF problem to a 3-DoF problem, significantly lowering computational complexity.
- **Computational efficiency:** Experiments show that our method achieves a single-solution time of only 0.142 ms, representing a 6× speedup compared to traditional three-view pose estimation methods. This efficiency advantage arises from the linearized solver design, which eliminates the need for iterative optimization and thus guarantees reliable real-time performance.
- **Robustness verification:** The method maintains strong stability under challenging conditions, including image noise (≤2 pixels), angular deviation (≤1°), and minor camera vibration. Notably, in the motion sequences of the KITTI dataset, the median rotational error is below 0.0545°, and the translational error is below 2.1319°, validating its applicability in real-world scenarios.

## 2. Related Work

### 2.1. Three-View Pose Estimation

Heyden et al. [6] proposed the trifocal tensor (TFT), which provides a theoretical framework for three-view geometry. Torr et al. [7] demonstrated that the tensor can be computed from six corresponding point triplets across three images. Indelman et al. [8] developed a vision-aided navigation method based on the geometry of three views, combining it with inertial navigation to estimate vehicle pose. Ponce et al. [9] introduced six homogeneous constraints and explored a novel approach to characterize the three-view problem. Ding et al. [5] investigated solving the problem of three views using four match points of feature points and demonstrated GPU acceleration for their HC solver, which requires an initial solution. Li et al. [10] introduced two novel solvers that incorporate vertical direction constraints from the IMU to estimate relative poses in the geometry of three views accurately. Although offering some performance enhancement, the general designs of these methods do not adequately exploit domain-specific motion constraints (e.g., planar motion), resulting in unnecessary computational overhead.

### 2.2. Planar Motion Pose Estimation

The problem of estimating the pose of the plane motion was first solved by Ortin and Montiel [11], who proposed two solvers to calculate the motion of the camera between two images: an iterative 2-point algorithm and a 3-point linear algorithm. Chou and Wang [12] developed a non-iterative 2-point algorithm based on an ellipse formulation to address image matching under significant viewpoint changes. Building on this work, Choi et al. [13] transformed the ellipse formulation into finding intersections between a line and a unit circle, proposing two non-iterative 2-point algorithms for relative pose estimation under planar motion. Guan et al. [14] proposed a novel minimal solution for planar motion estimation by utilizing constraints from image feature descriptors. While these methods mainly target two-view configurations, research on three-view pose estimation under planar motion remains relatively underdeveloped. Thus, developing efficient and robust three-view pose estimation methods for planar motion scenarios remains an unresolved research problem.

## 3. Model Building

### 3.1. The Projection Matrix Under Planar Motion

In 3D space, camera motion can be fully described by a rigid transformation comprising two components: rotation and translation. The rotation has 3 degrees of freedom (DOF), corresponding to rotations about the X-axis, Y-axis, and Z-axis, commonly parameterized as pitch, yaw, and roll angles, respectively. The rotation matrices $R_{x}$, $R_{y}$, and $R_{z}$ are defined as follows:

(1) $$R_{x}=\left[\begin{array}{ccc} 1 & 0 & 0\\ 0 & \cos\alpha & -\sin\alpha \\ 0 & \sin\alpha & \cos\alpha \end{array}\right],$$

(2) $$R_{y}=\left[\begin{array}{ccc} \cos\theta & 0 & \sin\theta \\ 0 & 1 & 0\\ -\sin\theta & 0 & \cos\theta \end{array}\right],$$

(3) $$R_{z}=\left[\begin{array}{ccc} \cos\beta & -\sin\beta & 0\\ \sin\beta & \cos\beta & 0\\ 0 & 0 & 1 \end{array}\right].$$

where α, θ, and β represent the camera’s pitch, yaw, and roll angles, respectively. The 3D rotation matrix R is the product of the three aforementioned rotation matrices:

(4) $$R=R_{x}R_{y}R_{z}.$$

The camera’s translational motion in general space also possesses 3 DOFs, corresponding to displacements along the X-axis, Y-axis, and Z-axis, which can be represented by a translation vector t:

(5) $$t={\left[\begin{array}{ccc} t_{x} & t_{y} & t_{z} \end{array}\right]}^{T},$$

where $t_{x}$, $t_{y}$, and $t_{z}$ represent the camera’s translations along the X-axis, Y-axis, and Z-axis, respectively. The translation vector explicitly captures the camera’s positional displacement, which, together with the rotation matrix, fully determines its rigid motion in 3D space.

The projection of a world point X to image point x is given by matrix P:

(6) x=PX.

The projection matrix P is given by

(7) P=K[R∣−Rt].

The matrix K denotes the camera calibration matrix, which contains intrinsic parameters such as focal length and principal point coordinates, and can be precisely obtained through camera calibration.

When the calibration matrix K is known, (6) can be expressed as

(8) $$K^{-1}x=K^{-1}PX.$$

This yields the normalized image coordinates $\hat{x}=K^{-1}x$ and the normalized projection matrix $\hat{P}=K^{-1}P$. The point $\hat{x}$ represents the image projection of the 3D world point X under a normalized camera model where the calibration matrix K equals the identity matrix I. After normalization, the projection model becomes independent of specific camera parameters. All computations are performed in a unified coordinate system, eliminating parameter variations across different camera models and enhancing the generality and robustness of the algorithm. For computational convenience, this paper employs normalized projection matrices and normalized image coordinates. The projection matrix $P_{k}$ can be expressed as

(9) $$P_{k}=\left[\begin{array}{cccc} 1 & 0 & 0 & 0\\ 0 & 1 & 0 & 0\\ 0 & 0 & 1 & 0 \end{array}\right]\left[\begin{array}{cc} R_{k} & -R_{k}t_{k}\\ 0 & 1 \end{array}\right],$$

where $R_{k}$ is the rotation matrix and $t_{k}$ the translation vector for the k-th view.

Mobile robots and autonomous vehicles exhibit characteristic planar motion in practical applications. A representative case is indoor navigation robots operating on ground surfaces, where the camera’s Y-axis remains normal to the ground plane, and motion between different views is restricted to Y-axis rotation and two-dimensional planar displacement. Under these constraints, the rotation matrix $R_{k}$ reduces to a simplified form dependent solely on the yaw angle θ:

(10) $$R_{k}=R_{y}=\left[\begin{array}{ccc} C_{y}^{k} & 0 & S_{y}^{k}\\ 0 & 1 & 0\\ -S_{y}^{k} & 0 & C_{y}^{k} \end{array}\right].$$

Among them, $C_{y}^{k}=\cos\left(\theta_{k}\right)$, $S_{y}^{k}=\sin\left(\theta_{k}\right)$. $\theta_{k}$ denotes the yaw angle of the *k*-th view. This simplification reduces the complexity of the rotation matrix, resulting in a more concise and efficient representation of rotation while maintaining consistency with the physical characteristics of planar motion.

Under planar motion, the camera’s Y-axis translation is always zero, and the translation vector $t_{k}$ simplifies to the following:

(11) $$t_{k}={\left[\begin{array}{ccc} t_{x}^{k} & 0 & t_{z}^{k} \end{array}\right]}^{T},$$

where $t_{x}^{k}$ and $t_{z}^{k}$ represent the translations along the X-axis and the Z-axis for the *k*-th view, respectively. This simplification accounts for the positional invariance along the camera’s Y-axis (normal to the motion plane) during planar movement, resulting in a more compact projection matrix representation.

Substituting Equations (10) and (11) into Equation (9) yields the planar motion projection matrix $P_{k}$ as

(12) $$P_{k}=\left[\begin{array}{cccc} C_{y}^{k} & 0 & S_{y}^{k} & -C_{y}^{k}t_{x}^{k}-S_{y}^{k}t_{z}^{k}\\ 0 & 1 & 0 & 0\\ -S_{y}^{k} & 0 & C_{y}^{k} & S_{y}^{k}t_{x}^{k}-C_{y}^{k}t_{z}^{k} \end{array}\right].$$

The projection matrix $P_{k}$ in Equation (12) represents the core expression under planar motion constraints. By incorporating the physical characteristics of practical camera motion and simplifying both rotation and translation components, it yields a compact form that directly facilitates the construction of linear equation systems.

### 3.2. Linear 3-Point Method

In three-view pose estimation, the first view’s coordinate system is assumed as the world reference frame, i.e.,

(13) $$R_{1}=I_{3\times 3},t_{1}={\left(0,0,0\right)}^{T}.$$

Here, $R_{k}$ and $t_{k}$ denote the rotation matrix and translation vector of the *k*-th view relative to the first view (*k* = 1, 2, 3). Using Equation (12), the projection matrices for all three views are expressed as

(14) $$P_{1}=\left[\begin{array}{c} I|0 \end{array}\right],P_{2}=\left[\begin{array}{c} A|a_{4} \end{array}\right],P_{3}=\left[\begin{array}{c} B|b_{4} \end{array}\right],$$

where A and B are 3 × 3 matrices, with vectors $a_{i}$ and $b_{i}$ denoting the *i*-th columns of the corresponding matrices (*i* = 1, …, 4).

In line with the foundational framework laid out by Hartley and Zisserman’s Multiple View Geometry in Computer Vision (2nd Edition, Chapters 15 and 16) [15], the trifocal tensor stands as a core mathematical entity in the geometry of three views, encapsulating the intrinsic geometric constraints between views. The trifocal tensor T constitutes a 3 × 3 × 3 tensor whose elements $T_{i}$ are derived through the following:

(15) $$T_{i}=a_{i}b_{4}^{T}-a_{4}b_{i}^{T},i=1,2,3.$$

In general, the trifocal tensor has 18 DOFs, a conclusion that can be derived through an analysis of the DOFs of projection matrices: The projection matrices $P_{1}$, $P_{2}$, and $P_{3}$ of the three views contain a total of 33 DOFs. Specifically, each 3 × 4 projection matrix itself has 12 elements. But due to scale ambiguity, the number of DOFs for each projection matrix is reduced by 1, so a single-view projection matrix has 11 DOFs. However, since the definition of the trifocal tensor is independent of the projective transformation of the world coordinate system (the choice of the world coordinate system does not alter the essential properties of the tensor), and the 3D projective transformation itself contains 15 DOFs, this redundant component needs to be excluded from the total degrees of freedom, ultimately yielding 18 independent parameters. This precisely corresponds to the number of independent parameters required for the trifocal tensor to describe the geometric relationships among three views [16,17].

**Figure 2:**
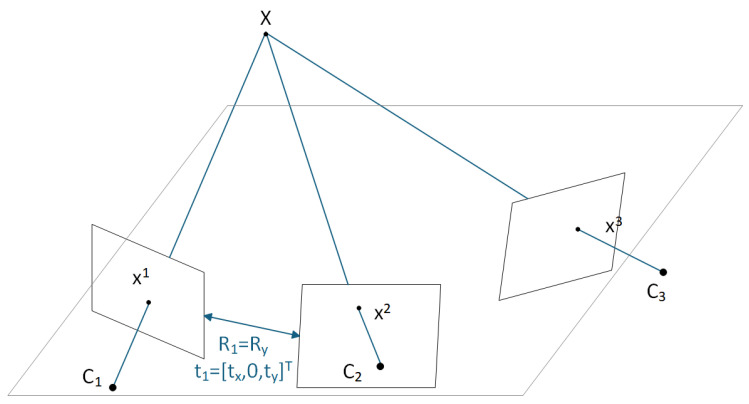
Point correspondences among three views under planar motion.

The simplified method for the tensor under planar motion proposed in this paper is an extension of classical theory under specific motion constraints: By reducing the DOFs of camera motion from 6 (3 rotational + 3 translational) to 3 (only rotation around the vertical axis + translation within the plane), the camera centers are strictly constrained within the X-Z plane. In this scenario, the normal vector of the plane formed by a spatial point and the three camera centers is fixed along the Y-axis (as shown in Figure 2). This stronger geometric constraint directly simplifies the tensor structure—the minimum number of corresponding point pairs required for solving is reduced from 7 to 3.

By substituting the simplified projection matrices Equations (12) and (14) into Equation (15), the specialized expression of the trifocal tensor under planar motion constraints is derived [10,18]:

(16) $$T_{1}=\left[\begin{array}{ccc} Q_{1} & 0 & Q_{2}\\ 0 & 0 & 0\\ Q_{3} & 0 & Q_{4} \end{array}\right]$$

(17) $$T_{2}=\left[\begin{array}{ccc} 0 & Q_{5} & 0\\ Q_{6} & 0 & Q_{7}\\ 0 & Q_{8} & 0 \end{array}\right]$$

(18) $$T_{3}=\left[\begin{array}{ccc} Q_{9} & 0 & Q_{10}\\ 0 & 0 & 0\\ Q_{11} & 0 & Q_{12} \end{array}\right]$$

It should be clarified that the planar motion constraint applies to the camera’s motion trajectory, specifically the position of the camera center, rather than the 3D points in the scene. While 3D points can still be located arbitrarily in space, as the camera centers are confined to the X-Z plane, the coplanar relationship formed between them and the spatial points is strengthened with a fixed normal vector. This enables the tensor parameters, which originally required more point correspondences for a solution, to be uniquely determined with fewer point correspondences—this constitutes the core logic of the simplified method.

To express the trifocal tensor more concisely, we introduce the symbol $\left\{\begin{array}{cccc} Q_{1} & Q_{2} & \ldots & Q_{12} \end{array}\right\}$ to denote Equations (16)–(18). The specific expression is as follows:

(19) $$\begin{array}{c} \left\{\begin{array}{cc} Q_{1}=C_{y}^{3}Q_{5}+C_{y}^{2}Q_{6} & Q_{2}=C_{y}^{2}Q_{7}-S_{y}^{3}Q_{5},\\ Q_{3}=C_{y}^{3}Q_{8}-S_{y}^{2}Q_{6}, & Q_{4}=-S_{y}^{3}Q_{8}-S_{y}^{2}Q_{7},\\ Q_{5}=C_{y}^{2}t_{x}^{2}+S_{y}^{2}t_{z}^{2}, & Q_{6}=-C_{y}^{3}t_{x}^{3}-S_{y}^{3}t_{z}^{3},\\ Q_{7}=S_{y}^{3}t_{x}^{3}-C_{y}^{3}t_{z}^{3}, & Q_{8}=-S_{y}^{2}t_{x}^{2}+C_{y}^{2}t_{z}^{2},\\ Q_{9}=S_{y}^{3}Q_{5}+S_{y}^{2}Q_{6}, & Q_{10}=C_{y}^{3}Q_{5}+S_{y}^{2}Q_{7},\\ Q_{11}=S_{y}^{3}Q_{8}+C_{y}^{2}Q_{6}, & Q_{12}=C_{y}^{3}Q_{8}+C_{y}^{2}Q_{7}. \end{array}\right. \end{array}$$

An examination of Equation (19) reveals the following findings:

(20) $$\left\{\begin{array}{c} Q_{10}\times Q_{8}-Q_{3}\times Q_{5}=\left(C_{y}^{3}Q_{5}+S_{y}^{2}Q_{7}\right)Q_{8}-\left(C_{y}^{3}Q_{8}-S_{y}^{2}Q_{6}\right)Q_{5}=S_{y}^{2}\left(Q_{7}Q_{8}+Q_{5}Q_{6}\right),\\ Q_{11}\times Q_{5}+Q_{2}\times Q_{8}=\left(S_{y}^{3}Q_{8}+C_{y}^{2}Q_{6}\right)Q_{5}+\left(C_{y}^{2}Q_{7}-S_{y}^{3}Q_{5}\right)Q_{8}=C_{y}^{2}\left(Q_{5}Q_{6}+Q_{7}Q_{8}\right),\\ Q_{11}\times Q_{7}-Q_{2}\times Q_{6}=\left(S_{y}^{3}Q_{8}+C_{y}^{2}Q_{6}\right)Q_{7}-\left(C_{y}^{2}Q_{7}-S_{y}^{3}Q_{5}\right)Q_{6}=S_{y}^{3}\left(Q_{7}Q_{8}+Q_{5}Q_{6}\right),\\ Q_{3}\times Q_{7}+Q_{10}\times Q_{6}=\left(C_{y}^{3}Q_{3}-S_{y}^{2}Q_{6}\right)Q_{7}+\left(C_{y}^{3}Q_{5}+S_{y}^{2}Q_{7}\right)Q_{6}=C_{y}^{3}\left(Q_{5}Q_{6}+Q_{7}Q_{8}\right),\\ C_{y}^{2}Q_{5}-S_{y}^{2}Q_{8}=C_{y}^{2}\left(C_{y}^{2}t_{x}^{2}+S_{y}^{2}t_{z}^{2}\right)-S_{y}^{2}\left(-S_{y}^{2}t_{x}^{2}+C_{y}^{2}t_{z}^{2}\right)=\left({\left(C_{y}^{2}\right)}^{2}+{\left(S_{y}^{2}\right)}^{2}\right)t_{x}^{2},\\ S_{y}^{2}Q_{5}+C_{y}^{2}Q_{8}=S_{y}^{2}\left(C_{y}^{2}t_{x}^{2}+S_{y}^{2}t_{z}^{2}\right)+C_{y}^{2}\left(-S_{y}^{2}t_{x}^{2}+C_{y}^{2}t_{z}^{2}\right)={\left({\left(S_{y}^{2}\right)}^{2}+{\left(C_{y}^{2}\right)}^{2}\right)}^{2}t_{z}^{2},\\ S_{y}^{3}Q_{7}-C_{y}^{3}Q_{6}=S_{y}^{3}\left(S_{y}^{3}t_{x}^{3}-C_{y}^{3}t_{z}^{3}\right)-C_{y}^{3}\left(-C_{y}^{3}t_{x}^{3}-S_{y}^{3}t_{z}^{3}\right)=\left({\left(S_{y}^{3}\right)}^{2}+{\left(C_{y}^{3}\right)}^{2}\right)t_{x}^{3}\\ -S_{y}^{3}Q_{6}-C_{y}^{3}Q_{7}=-S_{y}^{3}\left(-C_{y}^{3}t_{x}^{3}-S_{y}^{3}t_{z}^{3}\right)-C_{y}^{3}\left(S_{y}^{3}t_{x}^{3}-C_{y}^{3}t_{z}^{3}\right)=\left({\left(S_{y}^{3}\right)}^{2}+{\left(C_{y}^{3}\right)}^{2}\right)t_{z}^{3} \end{array}\right.$$

Under the condition that $({\left(C_{y}^{2}\right)}^{2}+{\left(S_{y}^{2}\right)}^{2})=1$ and $({\left(S_{y}^{3}\right)}^{2}+{\left(C_{y}^{3}\right)}^{2})=1$, we derive

(21) $$\begin{array}{c} \left\{\begin{array}{cc} \; & S_{y}^{2}=\frac{Q_{8}Q_{10}-Q_{3}Q_{5}}{Q_{5}Q_{6}+Q_{7}Q_{8}},\;S_{y}^{3}=\frac{Q_{7}Q_{11}-Q_{2}Q_{6}}{Q_{5}Q_{6}+Q_{7}Q_{8}},\;\\ \; & C_{y}^{2}=\frac{Q_{5}Q_{11}+Q_{2}Q_{8}}{Q_{5}Q_{6}+Q_{7}Q_{8}},\;C_{y}^{3}=\frac{Q_{3}Q_{7}+Q_{6}Q_{10}}{Q_{5}Q_{6}+Q_{7}Q_{8}},\\ \; & t_{x}^{2}=C_{y}^{2}Q_{5}-S_{y}^{2}Q_{8},\;t_{x}^{3}=S_{y}^{3}Q_{7}-C_{y}^{3}Q_{6},\\ \; & t_{z}^{2}=S_{y}^{2}Q_{5}+C_{y}^{2}Q_{8},\;t_{z}^{3}=-S_{y}^{3}Q_{6}-C_{y}^{3}Q_{7}. \end{array}\right. \end{array}$$

Considering a set of corresponding points $x^{1}↔x^{2}↔x^{3}$ in three views, the relationship between these corresponding points is as follows:

(22) $${\left[x^{2}\right]}_{\times }\left(\sum_{i}x_{i}^{1}T_{i}\right){\left[x^{3}\right]}_{\times }=0_{3\times 3},$$

where $x^{1}$, $x^{2}$, $x^{3}$ represent the image coordinates corresponding to View 1, View 2, and View 3, respectively, and $x_{i}^{1}$ denotes the *i*-th coordinate of $x^{1}$. Equation (22) contains nine equations, but rank analysis reveals that only four are independent. With 3-point pairs, we construct 12 equations, forming the following system:

(23) $$\left[\begin{array}{ccccc} h_{1}^{1} & h_{2}^{1} & \ldots & \ldots & h_{12}^{1}\\ h_{1}^{2} & h_{2}^{2} & \ldots & \ldots & h_{12}^{2}\\ \vdots & \vdots & \; & & \vdots \\ h_{1}^{12} & h_{2}^{12} & \ldots & \ldots & h_{12}^{12} \end{array}\right]\left[\begin{array}{c} Q_{1}\\ Q_{2}\\ \vdots \\ Q_{12} \end{array}\right]=0_{12\times 1}$$

The Equation (23) is in the form of Hq=0, where each element $h_{m}^{n}$ in H is known, with *n* representing the *n*-th equation and m representing the coefficient of $Q_{m}$. Therefore, $q={\left[\begin{array}{cccc} Q_{1} & Q_{2} & \ldots & Q_{12} \end{array}\right]}^{T}$ can be solved by SVD decomposition. Then $S_{y}^{k}$, $C_{y}^{k}$, $t_{x}^{k}$, $t_{z}^{k}$ can be solved by Equation (21), where *k* = 2, 3. Thus, the rotation matrix $R_{k}$ and translation vector $t_{k}$ can be determined, and the relative pose estimation between the three views can be obtained.

### 3.3. The Corrective Methods in Practical Applications

In practical applications, especially when dealing with real image data, to ensure the algorithm’s feasibility, we often need to correct the camera’s rotation matrix and translation vector first to make them meet the motion constraints of the plane. Before the correction, the camera rotation matrix between the first and second images is R, and the translation vector is t. After correction, the rotation matrix is $R^{\prime }$, and the translation vector is $t^{\prime }$. The following can be seen from Section 3.1:

(24) $$\left\{\begin{array}{c} R=R_{z}R_{x}R_{y},t={\left(\begin{array}{ccc} t_{x} & t_{y} & t_{z} \end{array}\right)}^{T},\\ R^{\prime }=R_{y},t^{\prime }={\left(\begin{array}{ccc} t_{x} & 0 & t_{z} \end{array}\right)}^{T}. \end{array}\right.$$

It is easily seen that $R=R_{z}R_{x}R^{\prime }$. Assuming the correction matrix $J=\left[\begin{array}{cc} R_{p} & T_{p}\\ 0 & 1 \end{array}\right]$, the following formula holds:

(25) $$\left[\begin{array}{cc} R & t\\ 0 & 1 \end{array}\right]=\left[\begin{array}{cc} R_{p} & t_{p}\\ 0 & 1 \end{array}\right]\left[\begin{array}{cc} R^{\prime } & t^{\prime }\\ 0 & 1 \end{array}\right].$$

Let us assume that $R_{p}=R_{z}R_{x}$, and we can solve $t_{p}=t-R_{p}t^{\prime }$. Thus, the correction matrix J can be solved.

After solving the correction matrix J, the image coordinates can be corrected so that the transformation relationship between the coordinates of different images is represented by the corrected rotation matrix $R^{\prime }$ and translation vector t. Assuming that the coordinates of the matching point in the first image are $X_{c1}$, the coordinates in the second image are $X_{c2}$, it can be introduced that

(26) $$X_{c2}=\left[\begin{array}{cc} R & t\\ 0 & 1 \end{array}\right]X_{c1}.$$

Substitute Equation (25) into Equation (26):

(27) $$J^{-1}X_{c2}=\left[\begin{array}{cc} R^{\prime } & t^{\prime }\\ 0 & 1 \end{array}\right]X_{c1}.$$

In other words, the corrected image coordinates satisfy $X_{c2}^{\prime }=J^{-1}X_{c2}$.

**Figure 3:**
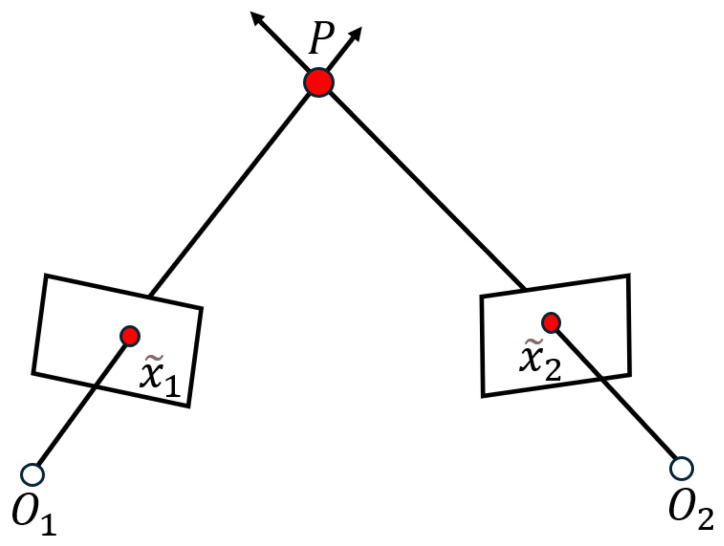
The principle of triangulation.

When correcting the image coordinates, we find that since the image coordinates used in the dataset are normalized coordinates ${\tilde{x}}_{2}$, and $X_{c2}=\left[\begin{array}{c} \lambda_{2}{\tilde{x}}_{2}\\ 1 \end{array}\right]$; the corrected image coordinates are $X_{c2}^{\prime }=J^{-1}\left[\begin{array}{c} \lambda_{2}{\tilde{x}}_{2}\\ 1 \end{array}\right]=\left[\begin{array}{c} R_{P}^{-1}\left(\lambda_{2}{\tilde{x}}_{2}-t_{p}\right)\\ 1 \end{array}\right]$. However, the depth information $\lambda_{2}$ of the matching points is unknown. To overcome this problem, we have adopted the triangulation method [15] to recover depth, as shown in Figure 3.

Triangulation refers to the observation of the same point P from different positions and inferring the coordinates of this point through the coordinates of the corresponding points in different images. Suppose ${\tilde{x}}_{1}$ and ${\tilde{x}}_{2}$ are the normalized coordinates of two corresponding points, ${\tilde{x}}_{1}={\left(\begin{array}{ccc} u_{1} & v_{1} & 1 \end{array}\right)}^{T}$, ${\tilde{x}}_{2}={\left(\begin{array}{ccc} u_{2} & \nu_{2} & 1 \end{array}\right)}^{T}$. The coordinates of point P in the world coordinate system are $P={\left(\begin{array}{ccc} x & y & z \end{array}\right)}^{T}$, as can be seen from the content of Section 3.1:

(28) $$\left\{\begin{array}{c} \lambda_{1}{\tilde{x}}_{1}=\left[\begin{array}{cc} R_{1} & t_{1} \end{array}\right]P=T_{1}P,\\ \lambda_{2}{\tilde{x}}_{2}=\left[\begin{array}{cc} R_{2} & t_{2} \end{array}\right]P=T_{2}P. \end{array}\right.$$

Decompose $T_{1}$ into row vectors $T_{11}$, $T_{12}$, and $T_{13}$, so we have:

(29) $$\lambda_{1}\left(\begin{array}{c} u_{1}\\ v_{1}\\ 1 \end{array}\right)=\left(\begin{array}{c} T_{11}\\ T_{12}\\ T_{13} \end{array}\right)P,$$

i.e.,

(30) $$\left\{\begin{array}{c} \lambda_{1}u_{1}=T_{11}P,\\ \lambda_{1}v_{1}=T_{12}P,\\ \lambda_{1}=T_{13}P. \end{array}\right.$$

In this system of equations, substitute $\lambda_{1}=T_{13}P$ into the first two equations:

(31) $$\left\{\begin{array}{c} \left(\lambda_{1}T_{13}-T_{11}\right)P=0,\\ \left(\lambda_{1}T_{13}-T_{12}\right)P=0. \end{array}\right.$$

Similarly, decompose $T_{2}$ into row vectors $T_{21}$, $T_{22}$, and $T_{23}$, and there is

(32) $$\left\{\begin{array}{c} \left(\lambda_{2}T_{23}-T_{21}\right)P=0,\\ \left(\lambda_{2}T_{23}-T_{22}\right)P=0. \end{array}\right.$$

The system of Equations (31) and (32) consists of four equations in the form of AP=0. The point P has three unknowns and can be calculated using the least square method.

## 4. Experimental Results and Analysis

This section presents extensive experiments on both synthetic data and real image sequences to validate the proposed method, with comparative analysis against existing algorithms in terms of computational efficiency, numerical stability, and noise robustness.

The proposed algorithm is designated as the Three-Point Method (abbreviated as Our-3pt). In our experiments, Our-3pt was systematically compared with the following classical algorithms: (1) Hartley-7pt, a general normalized solution for three-view problems (with its code available in [19]), which serves as our primary baseline; (2) Choi-2pt [13], a two-view method specifically designed for planar motion; and (3) two well-established two-view algorithms: Nister-5pt [20] and Hartley-8pt [15]. It should be noted that although Choi-2pt, Nister-5pt, and Hartley-8pt were originally designed for two-view problems, their mathematical frameworks can be extended to three-view relative pose estimation scenarios. This selection strategically covers both specialized planar solutions and general-purpose algorithms, facilitating multidimensional performance analysis. Our rigorous experimental protocol reveals fundamental insights into the efficacy of three-view geometric constraints for planar motion estimation, while objectively characterizing the method’s operational boundaries.

### 4.1. Simulated Data Experiment

#### 4.1.1. Computational Efficiency and Numerical Stability

Table 1 presents the average computation time of all algorithms after 5000 random trials on simulated data. All methods were tested under the same computer environment, equipped with an Intel(R) Core(TM) i9-14900HX 2.20 GHz processor and implemented in MATLAB R2023a. Experimental results in Table 1 demonstrate the superior computational efficiency of our proposed Our-3pt method. Specifically, the conventional three-view normalized solution Hartley-7pt requires an average runtime of 0.860 ms, which is over 6× longer than Our-3pt (0.142 ms). While the plane-specific Choi-2pt method achieves the shortest computation time (0.097 ms), it is limited to two-view scenarios and can only estimate a single camera pose. In particular, Our-3pt maintains an efficiency comparable to Hartley-8pt (0.183 ms) while simultaneously solving for two camera poses, demonstrating its practical advantage in planar motion estimation.

In our experimental evaluation, we employ two quantitative metrics to assess the performance of the algorithm: rotation error $\varepsilon_{R}$ and translation error $\varepsilon_{t}$. For rotation error, we compute the angular difference (in degrees) between the estimated and ground-truth rotation matrices. To address the scale ambiguity inherent in monocular vision systems, we similarly evaluate translation accuracy using angular deviation, specifically, the angle between the estimated and true translation directions (in degrees). The precise computational formulations follow those established in [21]:

(33) $$\left\{\begin{array}{c} \varepsilon_{R}=\arccos\left(\left(trace\left(R_{\mathrm{gt}}R^{T}\right)-1\right)/2\right)\\ \varepsilon_{t}=\arccos(\left(t_{\mathrm{gt}}^{T}t\right)/(∥t_{\mathrm{gt}}∥\cdot ∥t∥)) \end{array}\right..$$

where $R_{\mathrm{gt}}$ and $t_{\mathrm{gt}}$ denote the ground-truth rotation matrix and translation vector, while R and t represent their estimated values from the algorithm.

**Figure 4:**
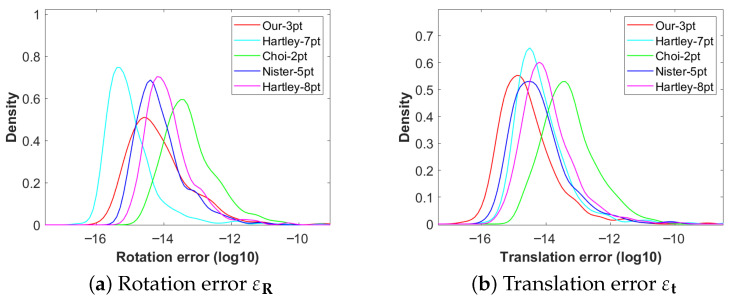
Probability density function of relative pose estimation error: (**a**) Rotation error (**b**) Translation error.

Without considering the influence of image noise, the experimental results of numerical stability are shown in Figure 4. All algorithms were executed 5000 times. The experimental results indicate that in terms of the estimation of the rotation matrix, Choi-2pt performs the best, followed by Our-3pt. Nister-5pt and Hartley-8pt show moderate performance, while Hartley-7pt is the worst. In terms of translation vector estimation, Our-3pt achieves the optimal performance, with Choi-2pt ranking second. Nister-5pt and Hartley-8pt still maintain a moderate level, and Hartley-7pt remains the worst. It is worth noting that Our-3pt not only excels in translation estimation but also maintains near-optimal performance in rotation estimation. The comprehensive evaluation demonstrates that Our-3pt exhibits significant overall advantages in numerical stability.

#### 4.1.2. Accuracy Analysis Under the Influence of Noise

**Figure 5:**
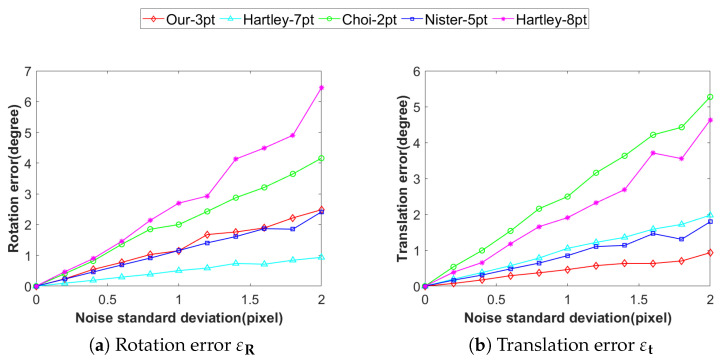
Accuracy under image noise: (**a**) rotation error, (**b**) translation error. (unit: degree).

To assess the accuracy and robustness of the proposed method against varying image noise levels, Gaussian noise (0–2 pixels) was introduced in simulations to replicate pixel-level errors arising from sensor noise and image processing during actual image acquisition. The experimental results are presented in Figure 5. It can be observed that the Our-3pt method exhibits significantly higher accuracy in solving the translation vector compared to the other four methods, characterized by both the smallest mean error and the least degree of error fluctuation. In terms of solving the rotation matrix, Choi-2pt achieves the best performance, while the precision of Our-3pt is almost comparable to that of Nister-5pt. In contrast, both Hartley-8pt and Hartley-7pt exhibit notable sensitivity to noise, with their errors increasing rapidly as noise levels grow.

**Figure 6:**
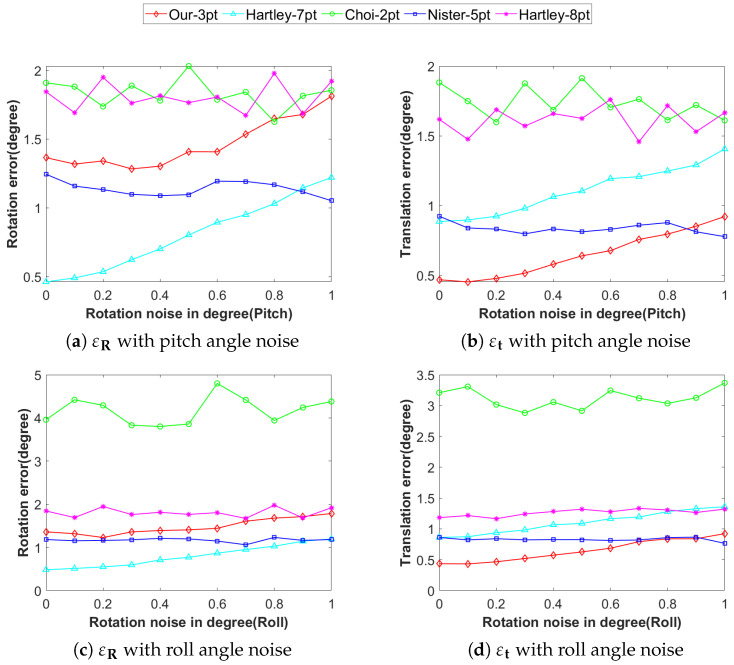
Accuracy under angular noise: (**a**) rotation error with pitch angle noise, (**b**) translation error with pitch angle noise, (**c**) rotation error with roll angle noise, (**d**) translation error with roll angle noise. (unit: degree).

In real-world scenarios, it is difficult to achieve ideal conditions. Although we assume that the camera only rotates around the y-axis, there may be some angular noise in practice on the x-axis and z-axis. The noise around the x-axis represents pitch angle noise, simulating the camera pitch jitter caused by the vehicle traveling up and down slopes or on bumpy roads. The noise around the z-axis represents roll angle noise, reflecting the camera’s roll deviation when the vehicle is turning or tilting. To investigate the impact of angular noise on algorithm accuracy, we set the angular noise range between 0° and 1°. The image noise is set to 1 pixel and remains unchanged. Since Hartley-7pt, Nister-5pt, and Hartley-8pt algorithms are designed for non-planar motion scenarios, they demonstrated relatively stable accuracy characteristics in the experiments. As shown in Figure 6, when the angular error is less than 1°, the translation accuracy and rotation accuracy of Our-3pt are superior to those of Hartley-8pt and Hartley-7pt. Compared with Nister-5pt and Choi-2pt, Our-3pt achieves the optimal performance in terms of translation accuracy, and its performance is significantly superior to that of Nister-5pt and Choi-2pt. Although in terms of rotation accuracy, Our-3pt fails to outperform Choi-2pt and Nister-5pt, its performance remains within an acceptable range.

#### 4.1.3. Accuracy Analysis Under the Influence of Vibration

**Figure 7:**
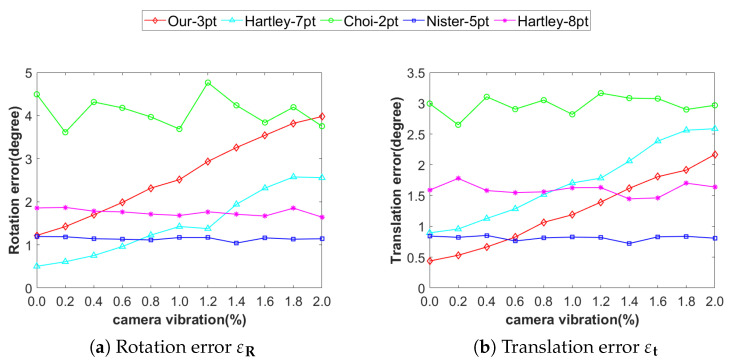
Accuracy under vibration effects: (**a**) rotational error, (**b**) translational error. (unit: degree).

During actual motion, the camera may experience minor vibrations along the Y-axis, preventing ideal planar motion. To evaluate the effect of these vibrations on the accuracy of the algorithm, we set their magnitude range to 0–1% of the displacement norm ($∥t∥$) between the current and initial positions, while maintaining a constant level of image noise of 1 pixel. As clearly observed in Figure 7, in terms of translation estimation, Our-3pt consistently outperforms Choi-2pt, demonstrating a stable precision advantage. Compared with Hartley-7pt, Our-3pt exhibits significant superiority in both rotation accuracy and translation accuracy. Compared with Hartley-8pt, Our-3pt achieves better rotation accuracy when vibration amplitude is below 0.4%$∥t∥$, while maintaining consistently higher translation accuracy within 0–1.4%$∥t∥$ vibration range. Although Nister-5pt outperforms Our-3pt in rotation accuracy, Our-3pt exhibits better translation accuracy when vibration remains below 0.6%$∥t∥$.

### 4.2. Real Image Sequences Experiment

#### 4.2.1. Comparison of Pose Estimation Accuracy in Real-World Scenarios

This section evaluates the proposed method on real-world image sequences from the KITTI dataset [22], with comprehensive performance comparisons against existing approaches. The KITTI dataset provides ground truth poses for 11 image sequences, obtained through high-precision GPS/IMU fusion. We evaluate performance using median errors per sequence. For fair comparison, all methods use standard RANSAC [23] without additional optimizations (e.g., inlier refinement, nonlinear optimization, or bundle adjustment [24]). All compared methods employ SIFT feature matching [23] to establish three-view geometric constraints under identical parameter thresholds. Our method is developed based on a planar motion assumption, which the KITTI dataset scenes do not strictly satisfy. Therefore, comparing its accuracy with other algorithms is not the primary objective of this experiment, as such a comparison would be inherently unfair to our method. Instead, this experiment aims to qualitatively verify whether the proposed method can be effectively applied to real-world scenarios. To address the discrepancy between the dataset and our assumptions, we preprocess the KITTI data through camera pose rectification and image coordinate transformation. This processing approximates planar motion conditions, enabling more valid experimental evaluation (refer to Section 3.3 for details).

The experimental results in Table 2 demonstrate that in terms of rotation estimation, as a three-view method, Our-3pt exhibits a significantly lower overall median rotation error compared to the classical three-view method Hartley-7pt, fully reflecting the optimization effect of the three-view model under planar motion constraints. When compared with other two-view methods, Choi-2pt performs optimally overall, with Our-3pt closely following. This trend is consistent with the simulation results, primarily because the three-view model needs to simultaneously constrain the geometric correlations among the three views, resulting in higher model complexity that may introduce minor cumulative errors. However, Our-3pt has already shown rotation accuracy close to that of Choi-2pt and outperforms Nister-5pt and Hartley-8pt.

The experimental results in Table 3 indicate that in terms of translation estimation accuracy, Our-3pt, as a three-view method, shows an overall performance comparable to the classical three-view method Hartley-7pt. In comparison with two-view methods, Nister-5pt achieves generally better translation estimation accuracy; nevertheless, Our-3pt demonstrates slightly superior translation accuracy in sequences 04, 06, and 07. In the remaining sequences, although the translation error of Our-3pt increases slightly, it remains within a reasonable range. Meanwhile, Our-3pt outperforms Choi-2pt in all test sequences and is generally superior to Hartley-8pt.

#### 4.2.2. Verification of Computational Efficiency and Feature Robustness in Real-World Scenarios

Table 4 presents a comparative analysis of the computational efficiency of the algorithms evaluated in the KITTI dataset, conducted under a unified RANSAC framework with standardized parameters. The framework utilizes the Sampson distance as the inlier criterion and adopts a 99% confidence level to ensure statistically robust sampling of minimal point sets. To balance efficiency and robustness, both the maximum number of iterations and the maximum number of sampling attempts are limited to 500. All compared algorithms share identical parameter configurations to ensure a fair evaluation. For runtime measurement, a robust two-stage statistical protocol is employed: first, the median execution time for each sequence is computed to mitigate the influence of outliers; subsequently, the final performance metric is derived as the mean of these median values across all 11 sequences, thereby ensuring reliable and representative timing comparisons.

Experimental results demonstrate that the proposed Our-3pt method achieves an average computational time of merely 1.26 ms on the KITTI dataset, exhibiting significant efficiency advantages. Compared to the conventional three-view Hartley-7pt approach (20.6 ms), our method demonstrates a remarkable 16.3× speedup, which convincingly validates the efficiency of our linearized solution framework based on planar motion constraints. Even when compared with the plane motion-optimized Choi-2pt method (1.66 ms), our approach maintains a 1.3× speed advantage while additionally providing the ability to estimate poses from three views. In broader algorithmic comparisons, our method shows 2.1× and 5.5× improvements in computational efficiency over Nister-5pt (2.70 ms) and Hartley-8pt (6.99 ms), respectively. These results conclusively demonstrate that our method successfully balances computational efficiency with estimation accuracy under planar motion assumptions, providing a viable technical solution for real-time applications such as autonomous driving where stringent timing requirements exist.

**Figure 8:**
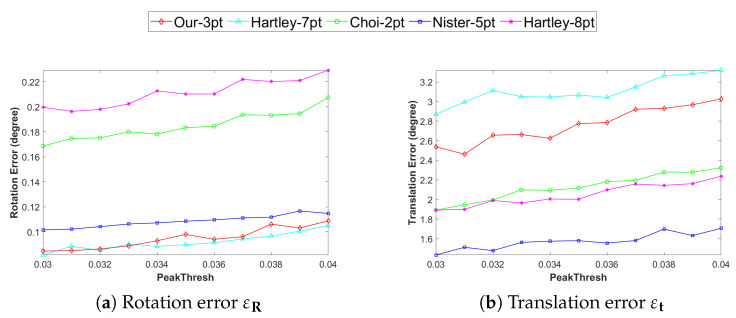
Pose estimation errors under different SIFT feature extraction thresholds: (**a**) rotational error, (**b**) translational error. (unit: degree).

To further verify the robustness of the proposed method in noise interference at feature points, this section is based on the sequence 00 of the KITTI data set. By adjusting the core parameter PeakThresh (peak threshold) for SIFT feature extraction, the impact of changes in the number of feature points on the pose estimation accuracy of five algorithms is explored. In the SIFT feature extraction process, the higher the PeakThresh value, the fewer the number of extracted feature points [25]. In the experiment, PeakThresh was gradually adjusted from 0.03 to 0.04 (with an interval of 0.01), and the rotation errors and translation errors of each algorithm were tested under different feature extraction accuracies. The results are shown in Figure 8.

The experimental results show that as PeakThresh increases and the number of feature points decreases, the accuracy of the pose estimation of different algorithms is significantly affected. In terms of rotation estimation accuracy, the Our-3pt and Choi-2pt methods perform the best, followed by Nister-5pt, and Hartley-7pt and Hartley-8pt perform the worst. In terms of translation estimation accuracy, Nister-5pt performs the best. The translation estimation error of Our-3pt is higher than that of Hartley-7pt and Hartley-8pt but lower than that of Choi-2pt. Overall, Our-3pt achieves a good balance between computational efficiency and accuracy, and is especially suitable for real-time pose estimation in planar motion scenarios.

## 5. Conclusions

This paper proposes a three-view relative pose estimation method based on planar motion constraints. By establishing trifocal tensor constraints and developing a linearized solution framework, the method achieves efficient pose estimation using only three point correspondences. Experimental results demonstrate superior accuracy, computational efficiency, and robustness in planar motion scenarios compared to existing approaches, providing a reliable solution for real-time pose estimation in mobile platforms such as autonomous vehicles, with significant practical applications. However, the limitation of this method lies in its strong dependence on the planar motion assumption: when there are significant non-planar motions in actual scenarios, the accuracy of pose estimation will decrease. Future work will explore multisensor fusion strategies (such as combining with IMU) to relax the planar motion constraints, and study robust feature screening mechanisms in dynamic environments to improve the applicability of the algorithm further.

## Figures and Tables

**Table 1:** Comparison of computation time for different methods (unit: ms).

Method	Our-3pt	Hartley-7pt	Choi-2pt	Nister-5pt	Hartley-8pt
Time	0.142	0.860	0.097	0.424	0.183

All time measurements are in milliseconds.

**Table 2:** Median rotational error of KITTI sequences (unit: degree).

Seq.	Our-3pt	Hartley-7pt	Choi-2pt	Nister-5pt	Hartley-8pt
00	0.0509	0.1190	**0.0380**	0.0866	0.1591
01	0.0537	0.4269	**0.0429**	0.0755	0.4803
02	0.0540	0.1331	**0.0383**	0.0887	0.1640
03	0.0545	0.1230	**0.0478**	0.0741	0.1674
04	**0.0285**	0.1469	0.0330	0.0762	0.1736
05	0.0347	0.1114	**0.0327**	0.0676	0.1414
06	**0.0330**	0.1154	0.0351	0.0672	0.1540
07	0.0362	0.1075	**0.0340**	0.0734	0.1390
08	0.0413	0.1204	**0.0333**	0.0798	0.1515
09	0.0406	0.1354	**0.0367**	0.0807	0.1704
10	0.0455	0.1226	**0.0349**	0.0794	0.1498

All values represent rotational error in degrees. Bold values indicate the best performance for each sequence.

**Table 3:** Median translation error of KITTI sequences (unit: degree).

Seq.	Our-3pt	Hartley-7pt	Choi-2pt	Nister-5pt	Hartley-8pt
00	1.5463	1.2917	1.8002	**1.2103**	1.4457
01	2.1319	2.8976	2.1547	**1.8397**	2.3018
02	1.3496	1.2865	1.6883	**1.1022**	1.3273
03	1.6843	2.0605	1.5764	**1.2532**	1.7406
04	**0.7774**	1.0534	1.4140	1.0244	1.2120
05	1.1093	1.5729	1.1420	**0.9484**	1.2034
06	**0.8023**	0.8500	1.4336	0.8147	1.0001
07	**1.3239**	1.4630	1.8569	1.3784	1.6365
08	1.6226	1.7043	1.4997	**1.3753**	1.6101
09	1.0632	1.6247	1.1977	**1.0043**	1.3199
10	1.5088	1.6111	1.3993	**1.1301**	1.4817

All values represent translation error in degrees. Bold values indicate the best performance for each sequence.

**Table 4:** Comparative runtime analysis of different methods under RANSAC framework on KITTI sequences (unit: ms).

Method	Our-3pt	Hartley-7pt	Choi-2pt	Nister-5pt	Hartley-8pt
Time	1.26	20.6	1.66	2.70	6.99

All time measurements are in milliseconds.

## Data Availability

The datasets analyzed in this study (KITTI sequences) are publicly available in the KITTI Vision Benchmark Suite repository: http://www.cvlibs.net/datasets/kitti/ (accessed on 11 August 2025). The specific implementation data and results generated during our experiments cannot be publicly shared due to technical constraints of our evaluation framework, but the complete methodological details are provided in the article to enable reproduction of our findings.

## References

[1] Chen C., Guan B., Shang Y., Li Z., Yu Q. (2023). A Ground Positioning Method with Rigidly Mounted IMU and Camera. Chin. J. Lasers.

[2] Gu M., Li H., Zhang J., Bai X., Zheng J. (2024). A Survey on Vision-Based UAV Positioning and Navigation Methods. Acta Electron. Sin..

[3] Gyagenda N., Hatilima J.V., Roth H., Zhmud V. (2022). A review of GNSS-independent UAV navigation techniques. Robot. Auton. Syst..

[4] Ma N., Cao Y.-F. (2024). A survey of vision-based perception and pose estimation methods for autonomous UAV landing. Acta Autom. Sin..

[5] Ding Y., Yang J., Ponce J., Kong H. Minimal solutions to relative pose estimation from two views sharing a common direction with unknown focal length. Proceedings of the IEEE/CVF Conference on Computer Vision and Pattern Recognition.

[6] Heyden A. (1997). Reconstruction from image sequences by means of relative depths. Int. J. Comput. Vis..

[7] Torr P.H.S., Zisserman A. (1997). Robust parameterization and computation of the trifocal tensor. Image Vis. Comput..

[8] Indelman V., Gurfil P., Rivlin E., Rotstein H. (2012). Real-time vision-aided localization and navigation based on three-view geometry. IEEE Trans. Aerosp. Electron. Syst..

[9] Ponce J., Hebert M. Trinocular geometry revisited. Proceedings of the IEEE Conference on Computer Vision and Pattern Recognition.

[10] Li T., Yu Z., Guan B., Han J., Lv W., Fraundorfer F. (2024). Trifocal Tensor and Relative Pose Estimation With Known Vertical Direction. IEEE Robot. Autom. Lett..

[11] Ortin D., Montiel J.M.M. (2001). Indoor robot motion based on monocular images. Robotica.

[12] Chou C.C., Wang C.-C. 2-point RANSAC for scene image matching under large viewpoint changes. Proceedings of the 2015 IEEE International Conference on Robotics and Automation (ICRA).

[13] Choi S., Kim J.-H. (2018). Fast and reliable minimal relative pose estimation under planar motion. Image Vis. Comput..

[14] Guan B.-L., Zhao J., Shang Y., Yu Q.-F. (2024). Minimal solutions for relative pose estimation under planar motion constraints. Sci. Sin. Technol..

[15] Hartley R., Zisserman A. (2003). Multiple View Geometry in Computer Vision.

[16] Lu L., Tsui H.T., Hu Z.Y. (2000). A novel method for camera planar motion detection and robust estimation of the 1D trifocal tensor. Proceedings of the 15th International Conference on Pattern Recognition, ICPR-2000.

[17] Chen J., Jia B., Zhang K. (2016). Trifocal tensor-based adaptive visual trajectory tracking control of mobile robots. IEEE Trans. Cybern..

[18] Guan B., Vasseur P., Demonceaux C. Trifocal tensor and relative pose estimation from 8 lines and known vertical direction. Proceedings of the 2022 IEEE/RSJ International Conference on Intelligent Robots and Systems (IROS).

[19] Julià L.F., Monasse P. A critical review of the trifocal tensor estimation. Proceedings of the Pacific-Rim Symposium on Image and Video Technology.

[20] Nistér D. (2004). An efficient solution to the five-point relative pose problem. IEEE Trans. Pattern Anal. Mach. Intell..

[21] Guan B., Zhao J., Barath D., Fraundorfer F. (2023). Minimal solvers for relative pose estimation of multi-camera systems using affine correspondences. Int. J. Comput. Vis..

[22] Geiger A., Lenz P., Stiller C., Urtasun R. (2013). Vision meets robotics: The KITTI dataset. Int. J. Robot. Res..

[23] Lowe D.G. (2004). Distinctive Image Features from Scale-Invariant Keypoints. Int. J. Comput. Vis..

[24] Triggs B., McLauchlan P.F., Hartley R.I., Fitzgibbon A.W. (1999). Bundle adjustment—A modern synthesis. International Workshop on Vision Algorithms.

[25] Scholkmann F., Boss J., Wolf M. (2012). An efficient algorithm for automatic peak detection in noisy periodic and quasi-periodic signals. Algorithms.

